# Collateral impact of the COVID−19 pandemic on the use of healthcare resources among people with disabilities

**DOI:** 10.3389/fpubh.2022.922043

**Published:** 2022-08-03

**Authors:** Minjeong Sohn, Heejo Koo, Heekyoung Choi, Hyunsan Cho, Euna Han

**Affiliations:** ^1^College of Pharmacy, Department of Pharmacy, Yonsei Institute of Pharmaceutical Sciences, Yonsei University, Incheon, South Korea; ^2^Division of Infectious Diseases, Department of Internal Medicine, National Health Insurance Service Ilsan Hospital, Goyang–si, South Korea; ^3^Institute of Human Genomic Study, Korea University Ansan Hospital, Ansan–si, South Korea; ^4^BK 21 Four R&E Center for Learning Health Systems, Korea University, Seoul, South Korea

**Keywords:** COVID−19, health services research, epidemiology, disability, healthcare use

## Abstract

**Objective:**

We assessed the collateral impact of the COVID−19 pandemic on healthcare service use among people with disabilities.

**Methods:**

We utilized the COVID−19 database from the Korean National Health Insurance Service claims from 2015 until June 2020. We included 5,850 people with disabilities and matched 5,850 without disabilities among those who were neither tested nor diagnosed with COVID−19. We used a quasi–experimental setting with a COVID−19 outbreak as an external event in a difference–difference estimation with matching controls.

**Results:**

Participants with disabilities recorded a larger decrease in the number of claims for total services (2.1 claims per 5 months) upon the COVID−19 pandemic's onset compared to those without disabilities (1.6 claims), and the difference–in–difference estimates were statistically significant (0.46 claims). The decline was driven by outpatient and emergency visits. The extent of the decline was large for the severe disability group overall. By disability type, those with a physical disability showed a statistically significant decline in the number of claims.

**Conclusion:**

The COVID−19 pandemic has had a collateral impact on people with disabilities' use of healthcare services. Continued assessment is needed regarding whether the collateral impact has been sustained or is following a different path.

## Introduction

The novel SARS–CoV−2 and the disease COVID−19 were first reported in late 2019, and the World Health Organization declared COVID−19 a global pandemic on March 11, 2020 (1, 2). The number of confirmed cases worldwide surpassed 1 million on April 2, 2020, 3 months after the first case was detected in central China (3). There were 360 million confirmed cases as of January 2022 (4).

The COVID−19 pandemic has caused collateral damage to the healthcare system in many countries; for example, the use of inpatient services fell by almost one–half after the onset of the pandemic in the United States, not only for elective surgeries but also for acute illnesses, such as stroke, cirrhosis, and myocardial infarction (5–8). The use of necessary services, such as vaccination or cancer treatment, has also declined during the pandemic (9, 10). Non–COVID−19 admissions in the United States declined between 39.5% and 50.0%, being more pronounced in poor and ethnic minority neighborhoods (11). By service type, emergency service use showed the largest decrease, followed by outpatient visits (12, 13). The decline in healthcare use might be due to a lower incidence of disease and a consequent low mortality rate, as in previous economic recessions (14), but it might stem from fear of infection, reduction in access associated with lockdown, and the cancellation of some elective services (12).

Considerable attention has been paid to the collateral influence of the COVID−19 pandemic on various non–COVID health outcomes, such as suicide (15), mortality, and healthcare utilization (16). However, these studies have mostly focused on the general population of a nation or a region. People with disabilities represent approximately 15% of the global population. The COVID−19 pandemic is likely to pose more challenges for people with disabilities than for the general population (17), considering that they may have limited access to information and communication, misconceptions, or administrative difficulties due to disruptions in assistive services (18–21). These challenges may increase in difficulty as people with disabilities are likely to be socioeconomically vulnerable: they are less likely to be employed, be reemployed, and have job security (22); they have weaker social networks (21); and they have lower disposable income due to extra costs associated with their disability (23). Together, these conditions raise concerns about the decrease in healthcare service utilization that might not have been so acute otherwise.

Health disparities—defined as avoidable differences in health status or healthcare (24)—between people with disabilities and the general population are a key public health issue. People with disabilities have reported more physical and mental health issues (25) and lower satisfaction with healthcare services (26). Financial constraints, secondary to the disability itself (27), could also contribute to reduced healthcare service use during the COVID−19 pandemic. This declining healthcare service use could exacerbate health disparities. This study explores whether people with disabilities use healthcare services less overall following the outbreak of COVID−19 and whether any variation exists by disability type and severity. We used a quasi–experimental approach and examined the COVID−19 pandemic onset in an event–study framework; people without disabilities were matched to people with disabilities to establish causality in the average collateral impact of COVID−19 on healthcare utilization among the latter group.

## Materials and methods

### Study sample

We analyzed data from the South Korean National Health Insurance Service (NHIS) COVID−19 database (DB), a retrospective cohort that includes all COVID−19 patients and their matched controls. The NHIS is the only public health insurer to have all Koreans as compulsory beneficiaries and every healthcare provider as a mandatory participant. The NHIS claims data include both enrollees' insurance qualification information and insurance claims from healthcare providers. Disability type and severity are included in the qualification information. In response to the COVID−19 pandemic, the Korean government publicly released the COVID−19 DB, which includes healthcare utilization data for 2020, covering medical claims from January 1, 2015, to July 31, 2020; this is the only publicly available dataset with healthcare use information from the COVID−19 pandemic period. The first diagnosis of COVID−19 in South Korea was made on January 8, 2020, so we considered 2020 as post–COVID−19 and all years up to 2019 as pre–COVID−19. Because the NHIS COVID−19 data were only compiled until July 2020, we used the 5–month data (from February to June) in each year from 2015 to 2020 for comparison.

The NHIS COVID−19 DB includes patients who were diagnosed with COVID−19 from January 1 to June 4, 2020, those who were tested for COVID−19 but not diagnosed; and those who were randomly selected to match the COVID−19 patients using a ratio of 1:15, matching by sex, age, and residential region. This study excluded COVID−19 patients and those tested for COVID−19 infection, noting that these groups might differ from the general population. The Korean government released a National Code of Conduct in March 2020 and ordered a mandatory quarantine for all confirmed cases and their close contacts, with the former being secluded in designated public hospitals with no out–of–pocket expenses (28). Therefore, we excluded the confirmed and tested cases in the study given that the healthcare use for the confirmed and test cases in the COVID−19 DB cannot be generalized, and our interest is to assess the collateral impact of the COVID−19 not the direct outcome of it.

**Figure 1:**
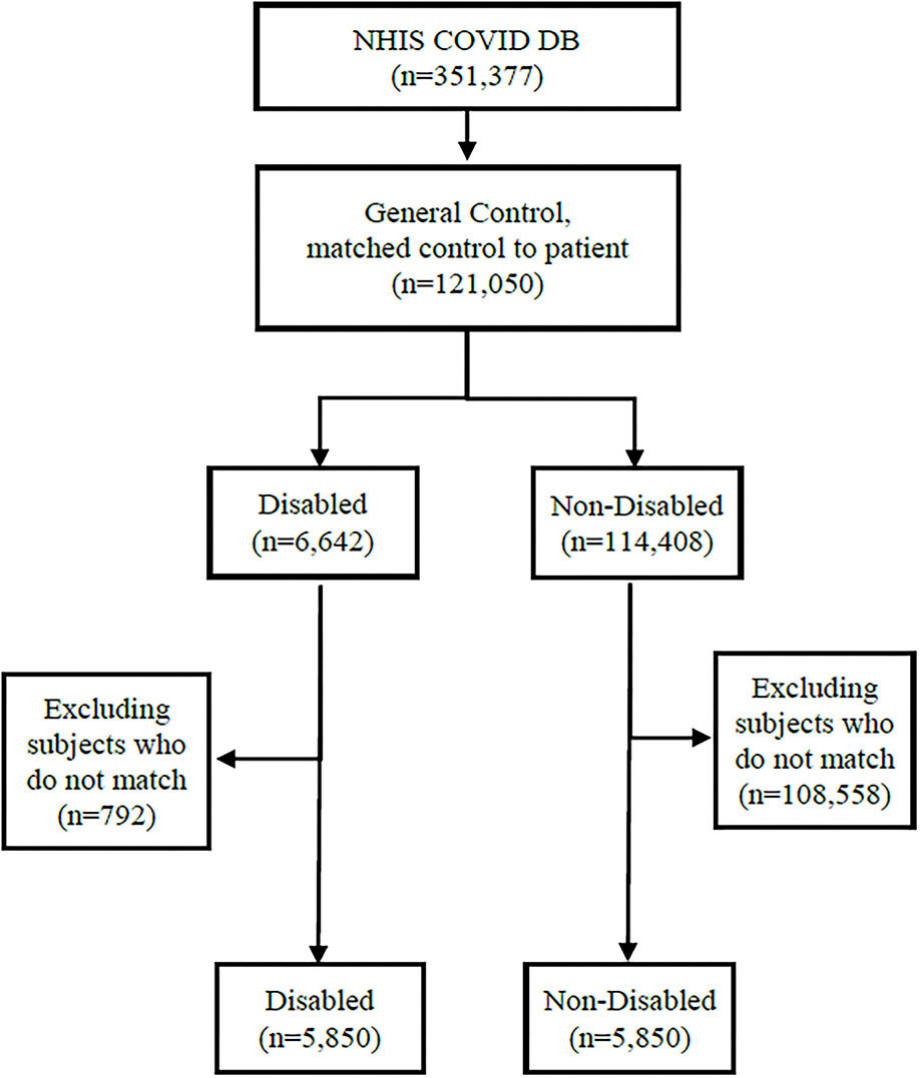
Sample selection flow.

Figure 1 presents the process of sample selection from the NHIS COVID−19 DB. Among the 351,377 individuals in the NHIS COVID DB, we identified those neither diagnosed nor tested for COVID−19 (*n* = 121,050) and classified them as disabled (*n* = 6,642) or nondisabled (n = 114,408). We matched the disabled and non-disabled groups using a 1:1 ratio, employing a propensity score matching method with age group, gender, and Charlson comorbidity index (CCI) as matching variables. Age was measured in 10–year intervals ranging from 0–9 years to 80 years or older. The CCI is a widely used composite indicator for comorbidities ranging between 0 (no comorbidity overall) and 16 (the highest level of comorbidity) for 19 diseases, each of which is weighted from 1 to 6 by severity (29). We grouped the combined CCI scores into 0, 1, 2, and 3 or higher for this study. We used 2019 as the index year for the CCI calculation. After the matching process, data from 5,850 participants with disabilities and 5,850 participants without disabilities remained for the analysis.

### Measures

The key independent variables were disability status, type, and severity. People with disabilities are required to register their physician's diagnosis with local governmental bodies to receive social welfare benefits (30). The insurance qualification database in the NHIS uses these registration data to compile disability information regarding type and severity, which is updated annually. We classified disabilities into the following categories: visual disability, hearing disability, physical disability, and others (including disability involving brain lesions; speech disability; intellectual disability; mental disorder; autistic disorder; kidney, cardiac, respiratory, or hepatic dysfunction; facial disfigurement; intestinal or urinary fistular; and epilepsy). We also used a binary classification for the presence of any disability. Disability severity is defined by the Enforcement Decree of the Act on Welfare of Persons with Disabilities, which has included two levels (mild or severe) since July 1, 2019, and included six levels (1 = most severe to 6 = most mild) before that point (31). For data from years before 2019, we collapsed grades 1–3 into the severe group and grades 4–6 into the mild group.

The outcome variables were total healthcare utilization and utilization by service type, including inpatient, outpatient, and emergency services in the insurance claims. Healthcare service utilization was measured as the total number of claims and total medical expenditures.

The covariates included qualification type, residential region, and income level. A linear variable corresponding to each year was controlled as the time trend. The qualification of health insurance was provided as insured as an employee, self–employed, and medical aid. The qualification information is a proxy for occupational status in this study. The residence was categorized into five regions.

### Analysis

We estimated a random–effects model in a difference–in–difference (DID) framework to assess COVID−19's impact on healthcare service utilization by disability status. This DID framework allowed us to compare COVID−19's effects on the dependent variables for the treatment group (people with disabilities) and the control group (people without disabilities), respectively, by controlling background changes in outcomes that occur with time (32). We used the following equation for the estimation:


$$\begin{array}{l} Y_{it}=\;\beta_{0}+\beta_{1}Disabled_{i}+\beta_{2}Post_{it}+Disabled_{i}\times Post_{it}+\beta_{4}X_{it}\\ \;+\beta_{5}Time_{t}+u_{i}+\varepsilon_{it}\; \end{array}$$

where *i* and *t* indicate each participant and each year, respectively. *Y* represents a series of dependent variables: healthcare utilization (medical expenditures and the number of claims) overall and by service type. If a specific service type was not used for a given observation, then the dependent variables were coded as zero. *Disabled* and *Post* are dummy variables denoting people with disabilities and the post–COVID−19 outbreak, respectively. *Time* is a linear variable that represents years, with 1 indicating the year 2015. *X* is a vector for the aforementioned confounding variables. *u*_*i*_ is a constant error component for each participant.

β_2_+β_3_ represents marginal changes in *Y* after the COVID−19 outbreak among people with disabilities compared to pre–outbreak. β_2_ represents the marginal change in *Y* after the COVID−19 outbreak among people without disabilities compared to pre–outbreak. Therefore, β_3_ is the DID estimate for the incremental change of *Y* after the COVID−19 outbreak among people with disabilities when the difference in *Y* between pre– and post–outbreak among people without disabilities is controlled.

We also estimated the DID after classifying disabilities as severe or mild as well as by disability type to assess variation in the COVID−19 outbreak's collateral impact by disability profile. All data extraction and statistical analyses were performed using SAS (version 9.4) and STATA (version 17).

## Results

Table 1 shows the study participants' general characteristics. Almost two–thirds of participants with a disability (64.6%) had a mild disability. Physical disabilities accounted for approximately half (44.1%) of total disabilities. More than half of all participants (i.e., both groups) were aged 60 or older. There were more than four times as many medical aid recipients among participants with disabilities (18.9%) compared to those without disabilities (4.6%) (Table 1).

**Table 1:** Summary statistics.

**Total**	**People without disabilities** ***N* (%)**	**People with disabilities** ***N* (%)**
	**5,850 (100.00)**	**5,850 (100.00)**
Severity of disability		
Severe	–	2,070 (35.4)
Mild	–	3,780 (64.6)
Disability type		
Physical	–	2,579 (44.1)
Visual	–	580 (9.9)
Hearing	–	947 (16.2)
Other	–	1,744 (29.8)
Gender		
Male	2,903 (49.6)	2,773 (47.4)
Female	2,947 (50.4)	3,077 (52.6)
Age group (years)		
0~9	14 (0.2)	14 (0.2)
10~19	44 (0.8)	44 (0.8)
20~29	433 (7.4)	435 (7.4)
30~39	218 (3.7)	221 (3.8)
40~49	397 (6.8)	405 (6.9)
50~59	1,094 (18.7)	1,112 (19.0)
60~69	1,703 (29.1)	1,459 (24.9)
70~79	1,291 (22.1)	1,197 (20.5)
80 or older	656 (11.2)	963 (16.5)
Charlson comorbidity index		
0	3,421 (58.5)	3,466 (59.3)
1	1,280 (21.9)	1,475 (25.2)
2	753 (12.8)	610 (10.4)
3 or higher	396 (6.8)	299 (5.1)
Qualification		
Self–employed	1,659 (28.4)	1,379 (23.6)
Salaried	3,920 (67.0)	3,361 (57.4)
Medical aid	271 (4.6)	1,110 (18.9)
Region		
Seoul	315 (5.4)	259 (4.4)
Kyunggi	3,840 (65.6)	3,850 (65.8)
Daegu	304 (5.2)	262 (4.5)
Kyungbook	872 (14.9)	940 (16.1)
Other	519 (8.9)	539 (9.2)

**Figure 2:**
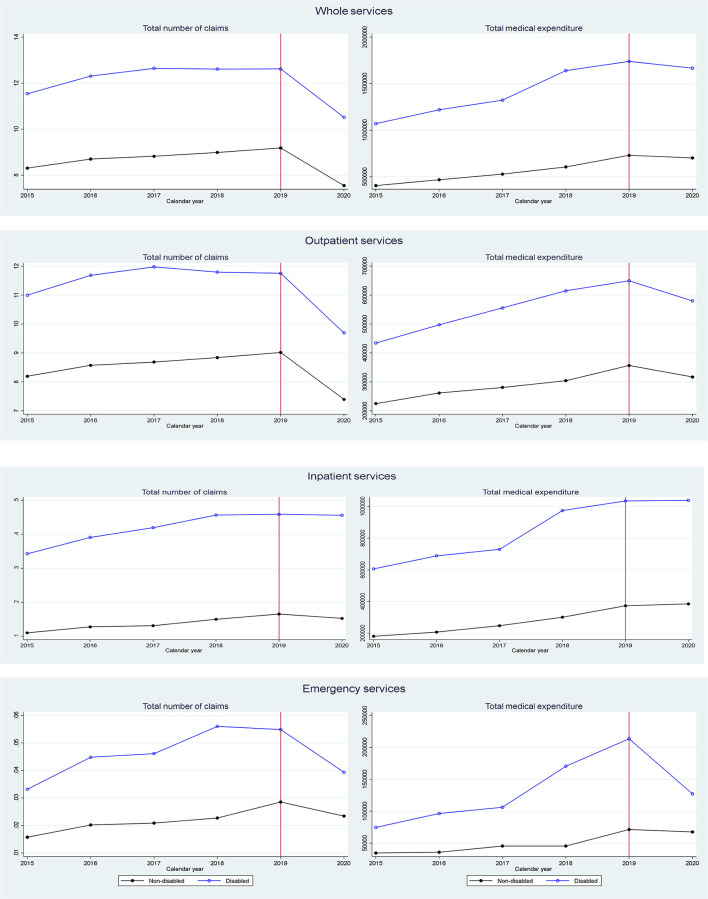
Average healthcare utilization trend for 5 months of each year pre- and post-outbreak of COVID-19.

Figure 2 shows the unadjusted yearly trend of healthcare service utilization between 2015 and 2020 for participants with disabilities compared to controls. There was a decline of approximately 10% in the total number of claims during the COVID−19 pandemic compared to the previous 5 years. Further examination by service type indicated that this decline at the beginning of the COVID−19 pandemic occurred mainly with respect to outpatient and emergency services, whereas the level of inpatient service utilization remained stable. There were also nearly parallel trends concerning total healthcare service utilization between participants with disabilities and the corresponding controls before the COVID−19 outbreak, which supports the DID framework's validity.

Dividing participants with disabilities into two groups by severity also uncovered an immediate decline in the number of claims at the beginning of the COVID−19 pandemic, with a larger decline for those with a mild disability compared to those with a severe disability. However, for emergency services, a sharper decrease was found among patients with severe disabilities than those with mild disabilities (Supplementary Figure 1). The yearly trends in healthcare service use by disability type also showed an approximately 10% decline in the number of claims for total, outpatient, and emergency services upon the outbreak across all disability types (Supplementary Figure 2).

Table 2 shows the results of the multivariate random–effects event analysis. To increase the analyses' efficiency within the sample, we estimated the average DID specifications for the aggregated pre–pandemic years. Participants with disabilities were estimated to have a larger decline in total healthcare service use (number of claims) upon the COVID−19 pandemic's onset compared to those without disabilities (2.1 vs. 1.6 claims per 5 months, respectively), and the difference–in–difference (0.46 claims) was statistically significant. Analyses by service type confirmed that this decline in the number of claims was driven by outpatient and emergency service use, as the DID estimate for each showed decreases of 0.56 and 0.01 claims per 5 months, respectively. Participants with disabilities also had a larger decline in medical expenditures after the outbreak compared to those without disabilities with respect to outpatient services (by KRW 69,224 and 39,501, respectively; 1 USD is approximately KRW 1,200) and emergency services (by KRW 42,102 and 39,061, respectively); however, the difference–in–differences in medical expenditures were not statistically significant for either service type.

**Table 2:** Multivariate random–effects regression of the incremental change in healthcare service utilization in the early COVID−19 pandemic compared to pre–pandemic for people with disabilities, controlling for the parallel difference among people without disabilities^d^.

**Service type**	**Expenditure**^**a**^ **(*****N*** = **11,700)**		***P*–value**	**Number of claims**^**a**^ **(*****N*** = **11,700)**		***P*–value**
	**b**	**(Standard error)**		**b**	**(Standard error)**	
Total^b^						
COVID−19 onset	−28,074	(46,796)	0.54	**−1.6357**	**(0.1220)**	<0.00
Disability	**861,515**	**(67,474)**	<0.00	**2.7963**	**(0.2431)**	<0.00
Disability × COVID−19 onset	−45,109	(66,179)	0.49	**−0.4643**	**(0.1725)**	0.00
Change between pre– and post–COVID−19 among nondisabled	−28,074	(46,796)	0.54	**−1.6357**	**(0.1220)**	<0.00
Change between pre– and post–COVID−19 among disabled	−73,183	(46,385)	0.11	**−2.1000**	**(0.1320)**	<0.00
Outpatient^b, c^						
COVID−19 onset	**−39,501**	**(13,665)**	0.00	**−1.8152**	**(0.1195)**	<0.00
Disability	**277,607**	**(30,157)**	<0.00	**2.5311**	**(0.1976)**	<0.00
Disability × COVID−19 onset	−29,723	(19,326)	0.12	**−0.5612**	**(0.1494)**	0.00
Change between pre– and post–COVID−19 among nondisabled before	**−39,501**	**(13,665)**	0.00	**−1.8152**	**(0.1195)**	<0.00
Change between pre– and post–COVID−19 among disabled	**−69,224**	**(13,085)**	<0.00	**−2.3764**	**(0.1185)**	<0.00
Inpatient^b, c^						
COVID−19 onset	11,427	(44,383)	0.79	−0.0128	(0.0148)	0.38
Disability	**557,402**	**(59,677)**	<0.00	**0.2143**	**(0.0216)**	<0.00
Disability × COVID−19 onset	−7,660	(62,767)	0.90	0.0104	(0.0210)	0.62
Change between pre– and post–COVID−19 among nondisabled before	11,427	(44,383)	0.79	−0.0128	(0.0168)	0.38
Change between pre– and post–COVID−19 among disabled	3,767	(44,834)	0.93	−0.0023	(0.0148)	0.87
Emergency^b, c^						
COVID−19 onset	**−39,061**	**(19,463)**	0.04	−0.0051	(0.0044)	0.24
Disability	**53,257**	**(8,063)**	<0.00	**0.0245**	**(0.0054)**	<0.00
Disability × COVID−19 onset	−3,041	(21,780)	0.88	**−0.0104**	**(0.0067)**	0.09
Change between pre– and post–COVID−19 among nondisabled before	**−39,061**	**(19,463)**	0.04	−0.0051	(0.0064)	0.24
Change between pre– and post–COVID−19 among disabled	**−42,102**	**(19,435)**	0.03	**−0.0155**	**(0.0044)**	0.00

^a^Number of individuals with disabilities and 1:1 matched control.

^b^Controlled for gender, age group, residential region, insurance qualification, and Charlson comorbidity index.

^c^For each service type, no corresponding service use was coded as zero.

^d^Statistically significant results at the 5% level are presented in bold.

^e^The covariates included qualification type, residential region, and income level. A linear variable corresponding to each year was controlled as the time trend.

^f^During the study period (January 1, 2015–July 31, 2020), USD 1 was equivalent to KRW between 1,065.67 and 1,210.80.

Table 3 shows the estimation results in relation to disability severity. Overall, participants with severe disabilities were estimated to have a larger decrease in healthcare service utilization compared to those with mild disabilities. Among participants with severe disabilities, total medical expenditures upon the onset of the COVID−19 pandemic were estimated to decline by KRW 91,534 for 5 months relative to the pre–COVID−19 period, controlling for the corresponding difference among participants without disabilities. Total medical expenditure for emergency services was also estimated to decline following the outbreak, with decreases being larger for those with severe disability relative to those with mild disability: KRW 132,054 vs. 61,340 for emergency services, respectively.

**Table 3:** Multivariate random effect regression of the incremental change in healthcare service utilization in early COVID−19 pandemic compared to pre–pandemic period for people with mild and severe disabilities compared to those without disabilities^d^.

**Service type**	**Expenditures^a^** **(*N* = 11,700)**	***P*–value**	**Number of claims^a^** **(*N* = 11,700)**	***P*–value**
	**b** **(Standard Error)**		**b** **(Standard Error)**	
Total^b^				
Severe disability × COVID−19 onset	**−91,534**	0.02	**−0.6066**	0.02
	**(42,552)**		**(0.2631)**	
Mild disability × COVID−19 onset	−74,692	0.17	**−0.6383**	0.00
	(46,510)		**(0.2147)**	
Inpatient services^b, c^				
Severe disability × COVID−19 onset	−56,745	0.53	0.0009	0.97
	(91,096)		(0.0314)	
Mild disability × COVID−19 onset	−66,926	0.36	−0.0242	0.34
	(74,335)		(0.0256)	
Outpatient services^b, c^				
Severe disability × COVID−19 onset	−33,542	0.17	−0.3728	0.14
	(29,508)		(0.2575)	
Mild disability × COVID−19 onset	**−27,631**	0.08	**−0.6006**	0.00
	**(15,789)**		**(0.2101)**	
Emergency visit^b, c^				
Severe disability × COVID−19 onset	**−132,054**	0.00	**−0.0371**	<0.00
	**(44,606)**		**(0.0086)**	
Mild disability × COVID−19 onset	**−61,340**	0.09	−0.0074	0.29
	**(36,398)**		(0.0070)	

^a^Number of individuals with disabilities and 1:1 matched control.

^b^Controlled for gender, age group, residential region, insurance qualification, and Charlson comorbidity index.

^c^For each service type, no corresponding service use was coded as zero.

^d^Statistically significant results at the 5% level are presented in bold.

^e^The covariates included qualification type, residential region, and income level. A linear variable corresponding to each year was controlled as the time trend.

There were different healthcare service utilization outcomes by disability severity in terms of the number of claims. Participants with mild disabilities showed a decline in the number of claims for total and outpatient services (by 0.6383 and 0.6066 per 5 months per year, respectively) compared to the pre–COVID−19 period, controlling for the corresponding difference among those without disabilities. For emergency services, only participants with severe disabilities had a lower number of claims (by 0.0371 per 5 months per year) compared to the pre–COVID−19 period, controlling for the corresponding difference among those without disabilities (Table 3, right panel).

Finally, participants with physical disabilities showed a significantly different change in the number of claims compared to those with other disabilities. Participants with physical disabilities showed a decline in the number of claims for total and outpatient services (by 0.5785 and 0.5541 per 5 months per year, respectively) upon the onset of the pandemic, controlling for the pre–post difference among those without disabilities (Table 4).

**Table 4:** Multivariate random–effects regression of the incremental change in healthcare service utilization in early COVID−19 pandemic compared to pre–pandemic period by disability type^d^.

**Disability subgroup**	**Expenditures**		***P*–value**	**Number of claims**		***P*–value**
	**b**	**(Standard error)**		**b**	**(Standard error)**	
Physical disability^a, b, c^ (*N* = 5,158)						
Total						
Disabled × COVID−19 onset	−28,261	(80,643)	0.72	**−0.5785**	**(0.2535)**	0.02
Inpatient						
Disabled × COVID−19 onset	−4,276	(74,688)	0.95	−0.0248	(0.0258)	0.33
Outpatient						
Disabled × COVID−19 onset	−24,041	(22,011)	0.27	**−0.5541**	**(0.2497)**	0.02
Emergency						
Disabled × COVID−19 onset	−15,550	(37,404)	0.67	−0.0031	(0.0087)	0.72
Visual disability^a, b, c^ (N = 1,160)						
Total						
Disabled × COVID−19 onset	−103,720	(182,940)	0.57	0.0810	(0.5401)	0.88
Inpatient						
Disabled × COVID−19 onset	−63,603	(168,167)	0.70	0.0275	(0.0371)	0.45
Outpatient						
Disabled × COVID−19 onset	−40,369	(51,711)	0.43	0.0517	(0.5368)	0.92
Emergency						
Disabled × COVID−19 onset	−62,009	(61,400)	0.31	−0.0069	(0.0152)	0.65
Hearing disability^a, b, c^ (*N* = 1,894)						
Total						
Disabled × COVID−19 onset	67,211	(149,744)	0.65	−0.3062	(0.4203)	0.46
Inpatient						
Disabled × COVID−19 onset	81,942	(143,871)	0.56	0.0063	(0.0464)	0.89
Outpatient						
Disabled × COVID−19 onset	−14,731	(31,113)	0.63	−0.3126	(0.4150)	0.45
Emergency						
Disabled × COVID−19 onset	−39,244	(69,719)	0.57	0.0105	(0.0139)	0.44
Other disabilities^a, b, c^ (*N* = 3,488)						
Total						
Disabled × COVID−19 onset	−111,523	(157,208)	0.47	−0.5625	(0.3314)	0.08
Inpatient						
Disabled × COVID−19 onset	−42,713	(151,267)	0.77	0.0590	(0.0522)	0.25
Outpatient						
Disabled × COVID−19 onset	−42,726	(50,597)	0.39	−0.4931	(0.3195)	0.12
Emergency						
Disabled × COVID−19 onset	−2,140	(57,589)	0.97	**−0.0338**	**(0.0138)**	0.01

^a^Number of individuals with each disability type and 1:1 matched control.

^b^Controlled for gender, age group, residential region, insurance qualification, and Charlson comorbidity index.

^c^For each service type, no corresponding service use was coded as zero.

^d^Statistically significant results at the 5% level are presented in bold.

^e^The covariates included qualification type, residential region, and income level. A linear variable corresponding to each year was controlled as the time trend.

## Discussion

The study revealed significantly larger decreases in healthcare service use overall and for outpatient and emergency services, particularly upon the onset of the COVID−19 outbreak among people with disabilities compared to those without disabilities. The study further showed that the severe disability group had a larger decline in medical expenditures compared to the mild disability group, with these declines being driven by emergency service use. The number of claims was estimated to decline for overall and outpatient services in the mild disability group but emergency services in the severe disability group.

Although our findings do not account for the underlying mechanisms driving such differences, they at least highlight the need for continuous scrutiny. People with disabilities confront more challenges in situations such as the COVID−19 pandemic. For example, non-transparent masks hinder communication for people with hearing disabilities (19). Studies have also reported substantial interruption of medical follow–up and rehabilitation during the lockdown for people with physical disabilities (33). Healthcare service disruption during the COVID−19 epidemic among people with disabilities and such disruption for chronic health conditions was also reported even in a sample with relatively higher socioeconomic status in the United States (34). Other than these administrative issues related to the pandemic, people with disabilities are more vulnerable to COVID−19 given their socioeconomic characteristics (35), which may impede the timely use of healthcare services. Social distancing during the pandemic has also led to restricted access to social welfare assistance for people with disabilities, which in turn may have reduced their utilization of routine healthcare services (17). People with disabilities are consequently likely to face increased marginalization in both routine and preventive care amid the pandemic. Thus, COVID−19's collateral impact on overall healthcare utilization could be stronger among people with disabilities. The decrease in the use of emergency services among people with severe disabilities could imply an interruption in healthcare services for those who require them most. Simultaneously, people with even mild disabilities must still be considered in public strategies to protect this demographic in social crises such as the COVID−19 outbreak (20, 21).

We estimated that the incremental decrease in the number of claims for overall healthcare services upon the onset of the COVID−19 pandemic was statistically significant among people with physical disabilities. There has been relatively few research on COVID−19's effect on people with disabilities (not to mention in terms of disability type) despite the significant attention paid to COVID−19's overall collateral impact (18). For example, a cross–sectional study including many US HMO patients reported that intellectual disability was the greatest risk factor for COVID−19 diagnosis and mortality (36). Two other studies investigating people with intellectual or developmental disabilities reported similar results regarding the COVID−19 fatality (37, 38). With people with disabilities' increased overall vulnerability during the pandemic, studies have also reported that certain disabled populations, such as those with visual or hearing impairments, may be less susceptible than others requiring routine medical follow–up and rehabilitation, such as those with physical or neurological disabilities (18, 39). Studies have suggested potential problems among people with hearing or visual impairments (40–43), although some studies do not provide supportive empirical evidence (42, 43). These arguments highlight the urgent need for data–informed strategies that address the heterogeneity in COVID−19's collateral impact due to disability characteristics. Researchers should pay close attention to people with disabilities when assessing any interruption of healthcare use during the pandemic, and more specific interventions targeting people with disabilities are required.

We acknowledge that this study has several limitations. First, some information is not present in insurance claims data because these data are originally collected administratively for reimbursement purposes. For example, in place of socioeconomic status, we were forced to substitute health insurance premium level, which is approximately proportionate to household incomes and assets, and qualification information for occupational status. We also note that the unit of analysis is claims per person rather than individual visits. Second, outpatient drug costs are not available in the COVID−19 DB. Third, we extracted the disabled and non-disabled groups from those who were neither tested for nor diagnosed with COVID−19 in the database. Nevertheless, this was the only database available in South Korea that included insurance claims during the COVID−19 outbreak.

Despite some caveats, this study has several merits. A recent study in Korea estimated that people with disabilities had higher risks of major adverse outcomes from COVID−19, including admission to the intensive care unit, invasive ventilation, and mortality (44). However, the researchers focused on confirmed COVID−19 patients to assess clinical outcomes among people with disabilities. Our study expands the investigation into the pandemic's spillover effect on people with disabilities in terms of healthcare service utilization. First, the national representativeness of the data is indisputable. We explored insurance claims data from the NHIS, to which every citizen in Korea is a mandatory subscriber and every medical provider is an obligatory participant. Second, the use of a quasi–experimental setting that exploited COVID−19 onset in an event–study framework is another merit of our study. We established the pandemic's causality by controlling for parallel pre–pandemic healthcare service use with a matched non–disabled group. Third, we identified heterogeneity in the pandemic's collateral effects in terms of disability severity and type. Additionally, we explored not only total healthcare service use but also use by service type.

Given the data's limited availability, we assessed only the immediate impact of the COVID−19 outbreak on healthcare service utilization. Whether this short–term impact will be sustained or follow a different path should be assessed further after the additional accumulation of data. Additionally, long–term studies should use more specific service characteristics for assessment—including preventive healthcare or targeted services, such as rehabilitation or assistive care—to enable more purposeful responses to similar future shocks for people with disabilities. Future studies also need to assess whether there are variations in the collateral impact of the COVID−19 pandemic on people with disabilities by socioeconomic or demographic characteristics as well as other disability profiles such as disability duration or types uninvestigated in this study.

Our findings have important implications for people with disabilities and the continuity of their care. People with disabilities used healthcare services (outpatient and emergency services, particularly) less frequently during the early stages of the COVID-19 pandemic relative to people without disabilities. Whether such declines stem from exacerbated access disparities during the COVID−19 outbreak and newly created disparities in health outcomes should be assessed in future work.

## Data availability statement

Publicly available datasets were analyzed in this study. This data can be found here: https://nhiss.nhis.or.kr/bd/ab/bdaba000eng.do;jsessionid=EdTiA5aI1e1JP24bLXzVxDO7AasuZvIoWYvEa1306FNRmmHU75Yx2DamU4FI8L4U.primrose2_servlet_engine10.

## Ethics statement

The studies involving human participants were reviewed and approved by Yonsei Institute Review Board (7001988– 202010–HR−1005–01E). Written informed consent for participation was not required for this study in accordance with the national legislation and the institutional requirements.

## Author contributions

EH conceptualized the study, designed methodology, and prepared original draft. MS wrote original draft. HK curated data, wrote original draft preparation and contributed visualization of the results. HC and HyC reviewed and revised the manuscript. All authors contributed to the article and approved the submitted version.

## Funding

Funding was provided by the National Research Foundation of Korea (Grant no. 2022R1A2B5B0100125311) and the National Evidence–Based Healthcare Collaborating Agency (Grant no. HC20C0010).

## Conflict of interest

The authors declare that the research was conducted in the absence of any commercial or financial relationships that could be construed as a potential conflict of interest.

## Publisher's note

All claims expressed in this article are solely those of the authors and do not necessarily represent those of their affiliated organizations, or those of the publisher, the editors and the reviewers. Any product that may be evaluated in this article, or claim that may be made by its manufacturer, is not guaranteed or endorsed by the publisher.
