# Supplementary material for: Collateral impact of the COVID−19 pandemic on the use of healthcare resources among people with disabilities
Source: Front Public Health. 2022 Aug 3;10:922043. doi: 10.3389/fpubh.2022.922043 (PMC9381991; doi:10.3389/fpubh.2022.922043)

Supplemental Figure 2. Average healthcare utilization trend for 5 months of each year by disability type pre- and post-outbreak of COVID-19

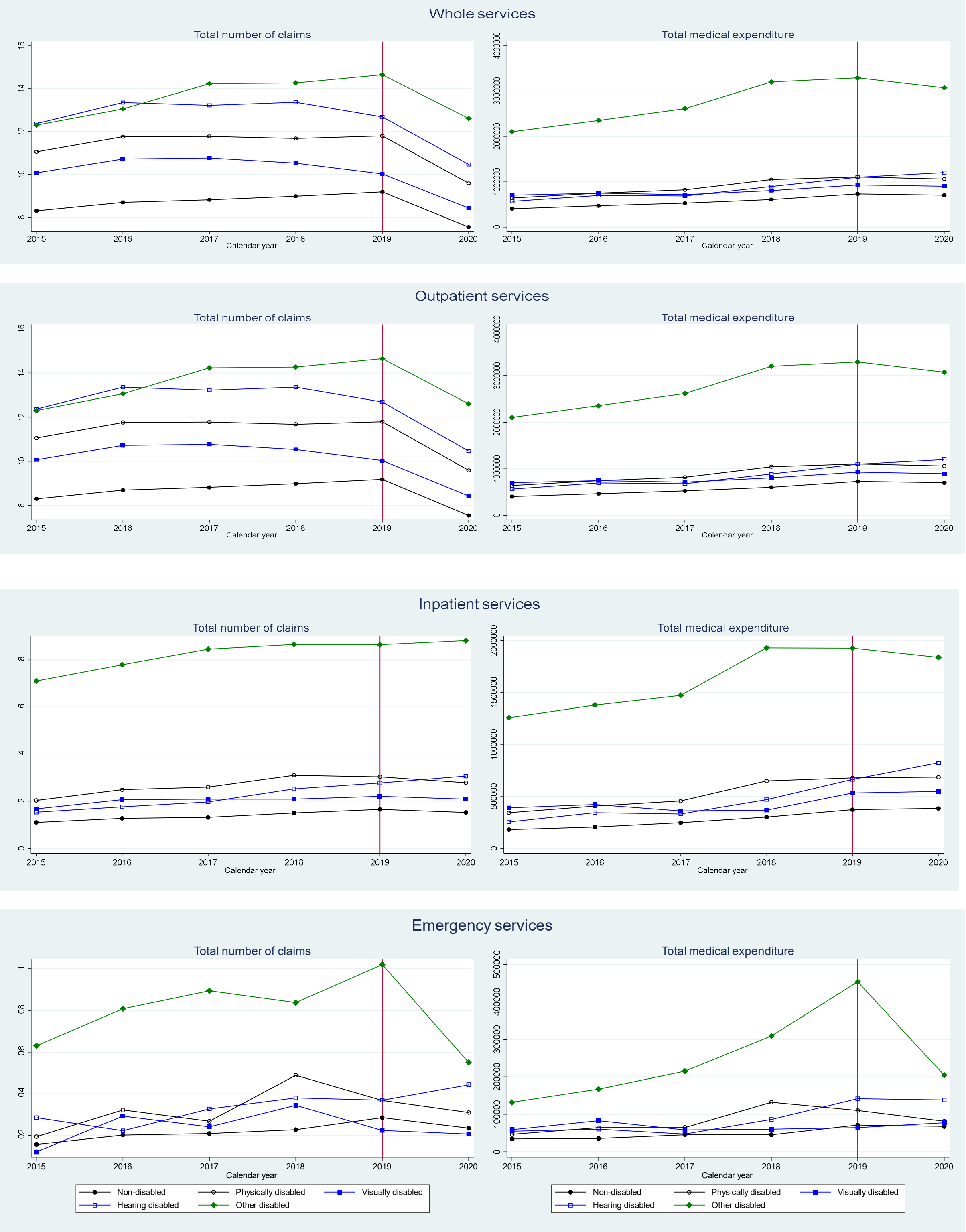

Supplement: Supplementary file 2 [file Data_Sheet_2.PDF]
